# Effects of Aging on Sucrose-Based Poly(ester-urethane)s: Thermal, Ultraviolet, and Hydrolytic Stability

**DOI:** 10.3390/polym18010088

**Published:** 2025-12-28

**Authors:** Violeta Otilia Potolinca, Cristian-Dragos Varganici, Florica Doroftei, Stefan Oprea

**Affiliations:** “Petru Poni” Institute of Macromolecular Chemistry, 41A Grigore Ghica Voda Alley, 700487 Iasi, Romania

**Keywords:** polyurethane, sucrose, thermal aging, UV irradiation, hydrolytic stability, mechanical properties, surface wettability

## Abstract

Environmentally friendly sucrose-based poly(ester-urethane)s were synthesized and characterized, and their stability and degradation behavior were assessed under three different aging conditions: thermal, ultraviolet (UV), and hydrolytic treatment. The specimens underwent thermal treatment in both hot and cold climates to simulate a temperate continental climate. The samples were thoroughly characterized to assess chemical and structural changes (FT-IR, TGA, and DSC) and surface modifications (contact angle measurements and AFM and SEM analyses), providing insights into surface morphology and wettability alterations. Mechanical testing was also performed to evaluate the retention rate of the strength and the elongation after the aging process. The results showed that the introduction of sucrose into the main chain of the polyurethanes protected the ester and urethane groups from environmental degradation. The best stability in all three degradation environments was achieved by PCL-poly(ester urethane) due to its higher degree of crystallinity. PCL-based polyurethane exhibited a fracture strength retention rate exceeding 85% under all aging conditions, while the weight ratio remained practically unchanged after hydrolytic degradation. Thus, the obtained polyurethanes may support the advancement of sustainable, eco-friendly materials for future industrial applications.

## 1. Introduction

Polyurethane is the most frequently used polymer for different applications. It is used in practically every part of our life, such as coatings, foams, adhesives, sealants, and composites [1,2,3,4,5]. It represents about 6% of the entire plastic production worldwide, ranking it as the 6th most popular polymer, with a market size estimated at USD 87.48 billion in 2025 and expected to reach USD 113.84 billion by 2030, registering a compound annual growth rate (CAGR) of 5.36% during the forecast period (2025–2030) [6,7]. Polyurethanes represent a key building block for modern material technologies and are exploited across a broad range of markets and applications that employ multiple raw materials [8]. The issue of polyurethane’s safe disposal has arisen as a result of increased production. Due to the irreversible deterioration of the environment caused by the use of non-renewable raw materials, the research focus on finding new sustainable feedstocks to replace the traditionally petroleum-based starting compounds in polyurethane production has significantly grown in the last century [9,10,11,12,13,14,15].

In addition to the environmental benefits of green polyurethanes, economic considerations are equally significant. The conventional production of polyurethanes relies heavily on petroleum feedstocks. While oil prices increase more and more and the availability of petroleum reserves is questionable, the use of carbohydrate derivatives (starch, chitin, chitosan, cellulose, lignin, etc.) and vegetable oils (castor oil, lesquerella oil, etc.) is attracting attention due to their low cost, biodegradability, high reactivity, and easy availability [16,17,18,19,20,21,22,23,24]. The introduction of polysaccharides into polyurethane chains leads to a material with a hydrophilic surface that provides a higher capability for degradation due to the adhesion of microorganisms to the surface, which promotes mass loss of both the natural and synthetic components of polyurethanes [25,26].

Carbohydrates are easily available renewable materials and contain numerous hydroxyl groups that are able to react with isocyanates, leading to urethane bonds. Among these, sucrose, a disaccharide, has been applied in polyurethane synthesis to achieve tailored material properties [27,28,29,30,31,32]. The biodegradation of linear and cross-linked sucrose- and glucose-based polyurethanes under solid and submerged fermentation and natural soil conditions revealed the superior biodegradability of sucrose-based polyurethanes [33]. Novel, well-defined sugar-based polyols were synthesized from methyl α-D-glucopyranoside, sucrose, and epoxidized methyl oleate [34]. Non-aromatic cross-linked polyurethanes based on disaccharides were developed as promising adhesive materials for medical applications [35]. Polyurethanes based on poly(ε-caprolactone)-diol, with sucrose serving as a chain extender and cross-linking agent, and hexamethylene diisocyanate were obtained and have potential applications in the biomedical and pharmaceutical domains [30]. Sucrose was also used in the synthesis of polyurethanes films and scaffolds to obtain potential tissue replacement materials [29]. Sucrose-based non-isocyanate polyurethane adhesives were obtained for plywood bonding as a sustainable alternative to traditional adhesives [36]. A copper oxide/polyurethane foam based on sucrose allows for efficient and sustainable solar-driven seawater desalination [37].

Weathering can deteriorate the physical and mechanical properties of polyurethane, limiting its service life in outdoor applications. The urethane and ether bonds are less susceptible to degradation; thus, most of the degradation tests are conducted on poly(ester-urethane)s [38,39]. Poly(ester-urethane)s can be used in many applications where degradation may occur, such as coatings, adhesives, and damping materials [40,41]. Cross-linked poly(ester urethane)/starch composite films were synthesized as eco-friendly food-packaging materials with increased durability [42]. Increased hydrolytic stability was achieved for a poly(ester amide urethane) developed from the α-amino acids phenylalanine and tryptophan as bio-derived building blocks [43]. Biodegradable poly(ester amide urethane) showed remarkable improvements in tensile strength characteristics after being exposed to different aging regimes, which supports their use as durable materials for outdoor applications [44].

To enable sustainable and scalable industrial processes, a major challenge lies in identifying low-cost, renewable building blocks that not only deliver performance benefits but are also derived from fungible feedstocks. The main aim of the current work is to synthesize new green polyurethane materials based on sucrose and different polyester diols and to study the influence of the environment (thermal aging, UV irradiation, and aqueous medium) on the physico-chemical characteristics to develop potential biocompatible materials with tailored properties. Sucrose may provide biodegradable properties to polyurethanes, while non-aromatic diisocyanate can improve their biocompatibility. By integrating natural raw materials at the stage of product design and development, the obtained polyurethanes can support the creation of more environmentally friendly materials for future industrial applications, promoting pollution prevention at the molecular level.

## 2. Materials and Methods

### 2.1. Materials

High-purity raw ingredients were used for the synthesis of the polyurethanes, and no further purification was needed. Different polyester diols, obtained from Sigma-Aldrich (ChemieGmbH, Taufkirchen, Germany) were used for the synthesis of the polyurethanes: poly(ethylene adipate) (PEA) (Mn = 2000 g mol^−1^), poly(diethylene glycol adipate) (PDEGA) (Mn = 2500 g mol^−1^), poly(hexamethylene carbonate) diol (PHMC) (Mn = 2000 g mol^−1^), poly(1,4 butylene adipate) diol (PBA) (Mn = 2000 g mol^−1^), and polycaprolactone diol (PCL) (Mn = 2000 g mol^−1^). Polyols were dehydrated for 2 h at 120 °C under high vacuum in order to eliminate any residual moisture. 1,6-Hexamethylene diisocyanate (HDI) was obtained from Fluka (Fluka Chemie AG, Buchs, Switzerland). Sucrose, dimethylformamide (DMF), and diiodomethane were obtained from Aldrich (Sigma-Aldrich, ChemieGmbH, Taufkirchen, Germany).

### 2.2. Sucrose-Based Polyurethane Synthesis

**Scheme 1:**
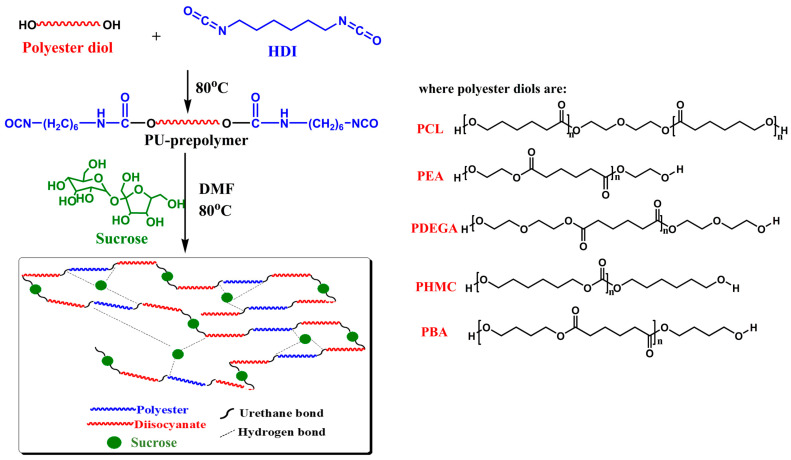
Schematic of the pathway for the synthesis of the sucrose-based poly(ester-urethane)s.

The polyurethane elastomers were synthesized by a previously reported procedure using the prepolymer method [45]. Briefly, a mixture of dehydrated polyester diol and a stoichiometric amount of HDI was added in a reactor at a 1:2 molar ratio and heated at 80 °C with vigorous stirring for 2 h. Sucrose has eight hydroxyl groups, but the reaction with the isocyanate occurs mainly on the three primary hydroxyl groups [30]. Thus, a predetermined amount of sucrose was dissolved in 10 mL DMF at 80 °C with stirring. After the complete dissolution of sucrose, the solution was added in one portion to the melted urethane pre-polymer previously obtained with vigorous stirring. The reaction mixture was kept at 80 °C for another 2 h until the disappearance of the isocyanate group absorption peak at around 2260 cm^−1^ in the FT-IR spectra. A schematic of the pathway for the synthesis of the sucrose-based poly(ester-urethane)s is illustrated in Scheme 1.

To obtain films, the polyurethane solutions were cast onto cleaned Teflon plates and placed in a vacuum oven at 80 °C for 24 h. Following preparation, the films were stored at room temperature for several days before being subjected to measurements. For the synthesis of the polyurethanes, different polyester diols were used, as shown in Table 1.

### 2.3. Thermal Aging

Due to the widespread application of polyurethanes in many different fields, it is essential to assess their longevity and durability. Thus, thermal aging of the specimens was performed using a SDJ8005 Programmable Low Temperature and Humidity Chamber (Shanghai Jianheng Instrument Co., Ltd., Shanghai, China). Investigating thermal, surface, and mechanical properties in low- and high-temperature environments is very important for a wide range of applications. Thus, the specimens were kept at 80 °C and −40 °C for one week using a heating/cooling speed of 2 °C/min. The extreme temperatures (80 °C and −40 °C) accelerate damage pathways, ensuring reliability in unexpected situations. After thermal treatment, the films were removed from the chamber and conditioned at room temperature for at least 40 h prior to testing.

### 2.4. UV Exposure

In order to study the stability of the sucrose-based poly(ester-urethane)s under UV light, the obtained films were exposed to UV irradiation using a SHH-150ZP UV Illumination Chamber (Shanghai Jianheng Instrument Co., Ltd., Shanghai, China) at a 320–400 nm wavelength range and a constant temperature (25 °C) for three weeks. The UV intensity was 210 μW/cm^2^ and the distance from the lamp was 10 cm. Following irradiation periods of one, two, and three weeks, the films were withdrawn from the chamber, conditioned at room temperature for a minimum of 40 h, and tested to assess the impact of UV exposure on the specimens’ properties.

### 2.5. Hydrolytic Degradation

The hydrolytic degradation of the obtained polyurethanes was carried out in distilled water at ambient temperature. At regular intervals, the film samples were removed, dried with filter paper, and weighed. The weight loss ratio (WL%) was calculated by the gravimetric method using the following formula:

(1) $$WL\%=\frac{m_{i}}{m_{0}}\times 100$$

where $m_{0}$ is the initial weight and $m_{i}$ is the weight at a specific time. The samples were analyzed every 30 days for a total of 600 days.

### 2.6. Characterization Methods

#### 2.6.1. Fourier Transform Infrared (FT-IR) Spectroscopy

The structure of the sucrose-based polyurethanes was confirmed using a Bruker VERTEX 70 instrument in attenuated total reflection (ATR; diamond crystal at 45°) mode for Fourier transform infrared spectroscopy (Bruker, Karlsruhe, Germany). The spectra were captured with a nominal resolution of 2 cm^−1^ in the infrared range of 4000–600 cm^−1^ at room temperature. FT-IR spectroscopy was also used to evaluate the changes upon UV/thermal aging.

#### 2.6.2. Thermogravimetric Analysis (TGA)

Thermogravimetric analysis (TGA) was carried out on a STA 449F1 Jupiter instrument (Netzsch, Selb, Germany) under a nitrogen flow of 50 mL/min and a heating rate of 10 °C/min. The measurements were performed from room temperature to 700 °C. The samples’ masses were around 10 mg.

#### 2.6.3. Differential Scanning Calorimetry (DSC)

DSC measurements were performed on a DSC 200 F3 Maia device (Netzsch, Selb, Germany), with a nitrogen flow rate of 50 mL/min and a heating/cooling rate of 10 °C/min. The thermograms were obtained from −100 °C to 250 °C. Polymers were analyzed during the second heating run. The mid-point temperature of the DSC signal’s change in slope was used to compute the glass transition temperature (T_g_) values of the polyurethanes. The weight of the samples was around 10 mg.

#### 2.6.4. Mechanical Analysis

Mechanical analysis was carried out on Shimadzu EZTest instrument (Shimadzu, Kyoto, Japan) equipped with a 5 kN load cell. The tensile properties were measured at ambient temperature (23 °C), with dumbbell-shaped specimens (ISO 37 Type 2) and a crosshead speed of 50 mm/min. Each sample was measured three times, and the average value was reported.

#### 2.6.5. Contact Angle and Surface Free Energy

A contact-angle optical goniometer (CAM-101, KSV Instruments, Helsinki, Finland) equipped with a video camera was used to measure static contact angles using the sessile drop method, and the results were evaluated at room temperature (23 °C). One microliter of liquid was applied to the polyurethane surface with a Hamilton syringe (Hamilton Company, Reno, Nevada). A minimum of five measurements were taken, and the average value is presented.

#### 2.6.6. Atomic Force Microscopy

An NTEGRA Spectra instrument (NT-MDT, Zelenograd, Moscow, Russia) with commercially available silicon nitride cantilevers (NSG10, NT-MDT, Zelenograd, Moscow, Russia) was used to perform atomic force microscopy (AFM) measurements. In semi-contact mode, 20 × 20 μm squares were scanned.

#### 2.6.7. Scanning Electron Microscopy

A Verios G4 UC Scanning Electron Microscope (SEM) (Thermo Scientific, Brno, Czech Republic) fitted with an Octane Elect Super SDD detector (Mahwah, NJ, USA) for Energy Dispersive X-ray spectroscopy analysis was used to assess the surface morphology. Using a Leica EM ACE200 sputter coater (Leica Microsystems, Vienna, Austria), the samples were coated with 10 nm platinum to enhance electrical conductivity and avoid charge accumulation when exposed to the electron beam. Using a secondary electron detector (Everhart–Thornley detector, ETD, Waltham, MA, USA) at an accelerating voltage of 5 kV, SEM studies were carried out in high vacuum mode.

## 3. Results and Discussion

### 3.1. Chemical and Structure Changes Measured by FT-IR Spectroscopy

**Figure 1:**
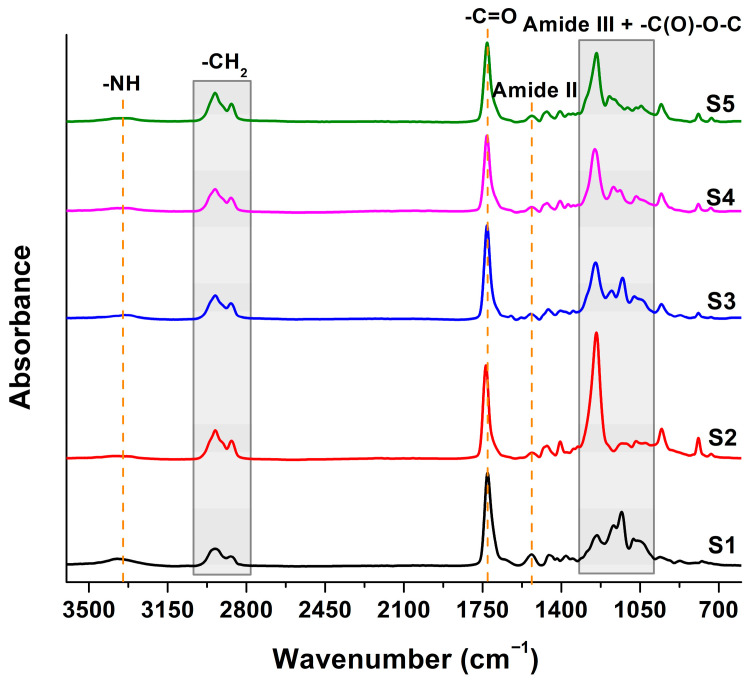
FT-IR spectra of the sucrose-based poly(ester-urethane)s.

The successful synthesis of the sucrose-based polyurethanes was evaluated by FT-IR spectroscopy, and the main specific bands are highlighted in Figure 1. Every spectrum displayed common bands associated with the structure of polyurethane: bands associated with the free and hydrogen-bonded NH stretching vibration in the range of 3700–3200 cm^−1^, asymmetric and symmetric stretching vibrations of the methylene and methyl groups in the range of 3000–2800 cm^−1^, free and hydrogen-bonded C=O stretching vibration (amide I) in the range of 1780–1650 cm^−1^, amide II (the stretching vibration of the C-N bond and the deformation vibration of the C-N-H bonds) in the range of 1600–1500 cm^−1^, amide III (the deformation vibration of the N-H bond and the deformation vibration of the O-C-N bonds) in the range of 1300–1200 cm^−1^, and the bands corresponding to the -C(O)-O-C stretching vibration assigned to the soft segment of the polyurethane and to the cyclic structure of disaccharide for sucrose can be observed in the range of 1200–990 cm^−1^ [31].

#### 3.1.1. Structural Changes After Thermal Aging

**Figure 2:**
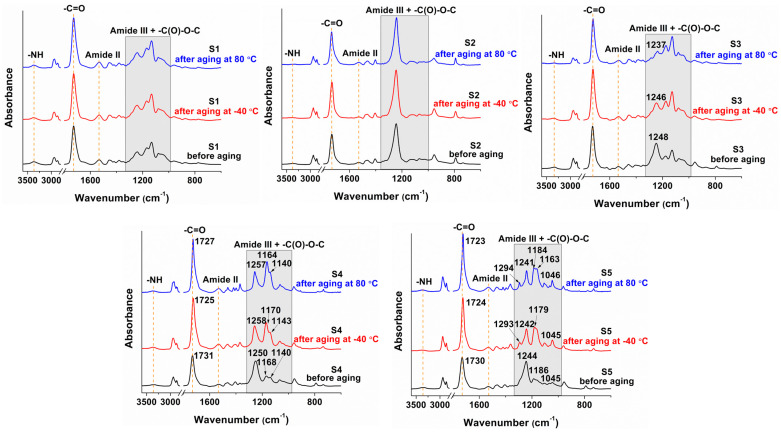
FT-IR spectra of the sucrose-based poly(ester-urethane)s before and after thermal treatment.

The changes in the structures of the obtained poly(ester-urethane)s after thermal aging were not significant and the samples kept their structural integrity, as shown in Figure 2. The polyurethanes S4 (with poly(1,4 butylene adipate) diol as soft segments)) and S5 (with polycaprolactone diol as soft segments) underwent slight changes in the amide I and amide III regions. The -C=O urethane bands shifted from ≈1730 cm^−1^ to ≈1725 cm^−1^ after the thermal treatment. This suggests enhanced formation of hydrogen-bonded urethane groups. These changes were corroborated with increases in the amide III and -C(O)-O-C bands, which confirmed the formation of a network with a higher density of inter- and intramolecular hydrogen bonding.

#### 3.1.2. Structural Changes After UV Irradiation

**Figure 3:**
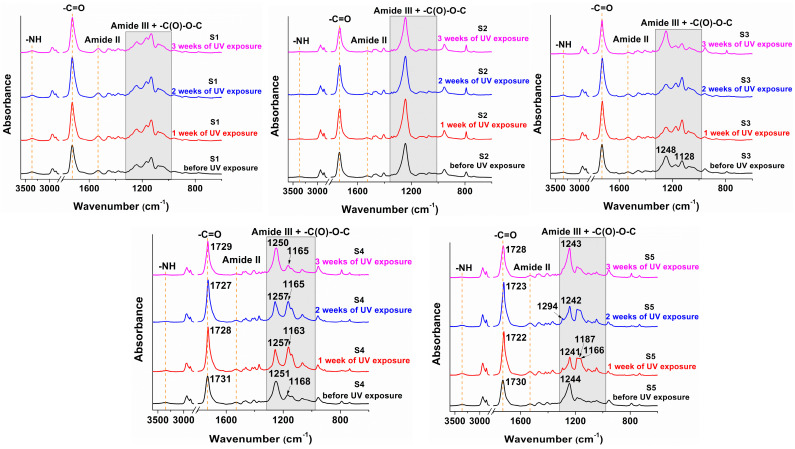
FT-IR spectra of the sucrose-based poly(ester-urethane)s before and after UV treatment.

After up to three weeks of UV exposure (Figure 3), the structures of the polyurethanes remained relatively unchanged, indicating that these formulations may confer structural stability under weathering conditions. The use of PBA (S4) and PCL (S5) as soft segments led to small changes in the 1300–1000 cm^−1^ range. There was an increase in the intensity of the -C(O)-O-C bands, suggesting a rearrangement of the macromolecular chains and leading to a more defined network. However, there was a slight decrease in the hydrogen bonded -NH stretching vibration in the range of 3700–3200 cm^−1^ which indicated some loss of the urethane structure [46].

Overall, the introduction of sucrose into the main chain of the polyurethanes protects urethane groups from environmental degradation, including heat, cold, and UV exposure. Slight changes in absorption peak intensities and spectral positions were observed, indicating limited structural damage.

### 3.2. Thermogravimetric Analysis

**Figure 4:**
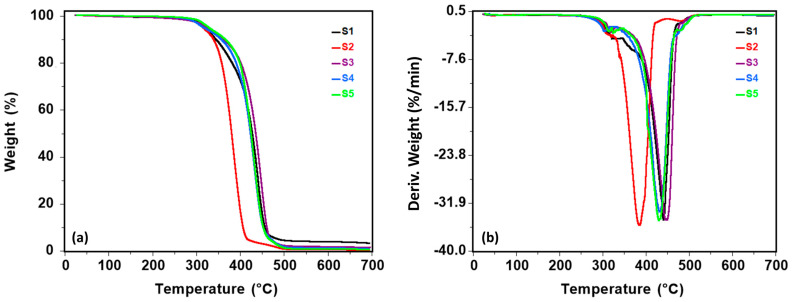
TG (**a**) and DTG (**b**) curves of the sucrose-based poly(ester-urethane)s.

Thermogravimetric analysis of the obtained polyurethanes was performed to assess the influence of the polyester type on thermal decomposition processes. The selection of suitable polyesters to improve thermal stability is a key factor in the development of future high-performance materials. Thus, the thermogravimetric curves of the sucrose-based poly(ester urethane)s are depicted in Figure 4.

All polyurethanes presented good thermal stability with a 5% mass loss at temperatures over 315 °C. The polyester type does not significantly influence the initial degradation temperature. The highest stability was achieved for S5 polyurethane (T_5%_ = 328 °C) with PCL as the soft segment. All samples showed three stages of degradation, and the characteristics of each stage are summarized in Table 2. The first stage corresponds to the decomposition of the urethane bonds from the hard segments. The second stage is associated with the scission of the soft segments, and the last stage is related to the decomposition of some recombined fragments [47,48]. The main degradation process is observed in the second step, where weight loss exceeded 70%. Surprisingly, the lowest thermal stability (T_5%_ = 315 °C and T_max_ = 384 °C) was obtained for S2 polyurethane, which contains poly(hexamethylene carbonate) diol. Poly(hexamethylene carbonate) diol should impart better thermal stability due to the carbonate linkage, which restricts segmental mobility and enhances dimensional stability. However, sucrose incorporation in the second reaction step disrupted network integrity, concealing the expected effect and lowering the thermal stability relative to other polyester urethanes. The highest T_max_ value was obtained for S3 polyurethane (447 °C), which was synthesized with poly(diethylene glycol adipate). Char residues found at 700 °C varied from 0.47% to 3.35%. The lowest percentage (0.47%) was obtained for S2 polyurethane, in line with its reduced thermal stability.

### 3.3. DSC Study

**Figure 5:**
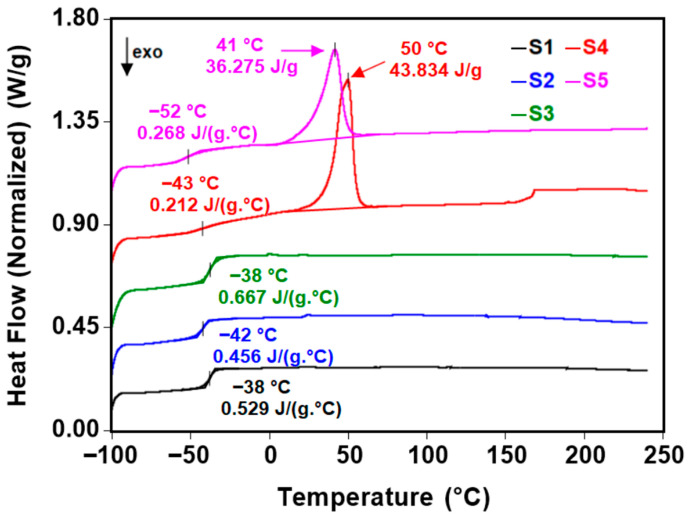
DSC thermograms of the sucrose-based poly(ester-urethane)s.

The DSC approach is a handy and effective method for experimental characterization, as it can determine the glass transition, melting, and crystallization temperatures of polymeric materials. It can be used to monitor the modification of the transition after aging. DSC thermograms from the second heating run are shown in Figure 5, and the corresponding thermal parameters are included in Table 3. The different polyester diols had a significant role in the glass transition temperature. The T_g_ values associated with the soft segments, i.e., polyesters, increased from −52 °C (for S5) to −38 °C (for S1 and S3 samples). The rigid backbone of poly(ethylene adipate) and poly(diethylene glycol adipate) raised the T_g_ of the polyurethanes, restricting chain mobility. Differences in chain mobility, dictated by the polyester diol type, are also mirrored in the heat capacity trend. Indeed, a decrease in the heat capacity values from 0.5–0.6 J g^−1^ C^−1^ (S1 and S3) to 0.2 J g^−1^ C^−1^ (S4 and S5) was observed.

S4 and S5 polyurethanes presented endothermic peaks at 50 °C and 41 °C, respectively, due to their higher crystallinity compared to the S1, S2, and S3 samples. This process is associated with the melting of the soft segments. The melting enthalpy is higher when PBA was used as a polyester diol (43.834 J g^−1^ for S4 vs. 36.275 j g^−1^ for S5).

#### Effects of Thermal and UV Aging on Crystallization and Melting Behavior

The polymers’ melting temperature (T_m_) may vary during aging, as molecular changes in the matrix affect the crystalline structure. Shifts in the glass transition temperature, caused by concurrent cross-linking or chain scission, reflect the effects of aging through changes in polymer chain mobility and rearrangements. Branching in polyesters could also increase these values [49].

**Figure 6:**
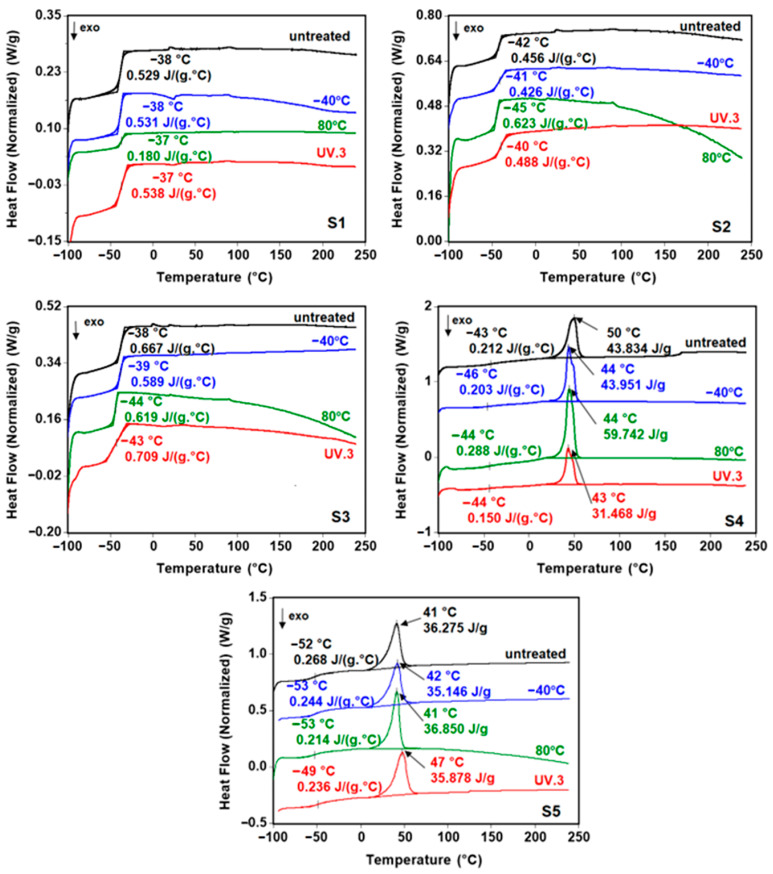
DSC thermograms of the sucrose-based poly(ester-urethane)s: untreated, thermally aged (−40 °C and 80 °C), and UV irradiated (UV.3).

Figure 6 shows the DSC curves of the sucrose-based poly(ester urethane)s before and after thermal aging and three weeks of UV irradiation (UV3). The thermal parameters are presented in Table 3. The thermal parameters of both pristine and aged polyurethanes changed slightly, suggesting negligible impacts of thermal and UV exposure on their thermal behaviors. The soft segments’ melting temperature had a more substantial variation because the crystalline structure of the molecules was altered by molecular modifications inside their matrix. The use of PBA polyester (S4) led to a decrease of 20% in the T_m_ values after the aging process (thermal or UV irradiation), from 50 °C to 43–44 °C. Instead, PCL (S5) increased the melting temperature of the soft segments of the corresponding polyurethane by 15% upon UV irradiation, from 41 °C to 47 °C.

### 3.4. Mechanical Properties

The nature of the polyol plays a significant role in determining the characteristics of polyurethanes. Mechanical properties are dictated by the structure, molecular weight, and morphology. The use of sucrose, a disaccharide, leads to polyurethanes with low mechanical parameters due to a destabilization of the cohesion between molecular chains. Similar results have been achieved previously [27,31].

**Figure 7:**
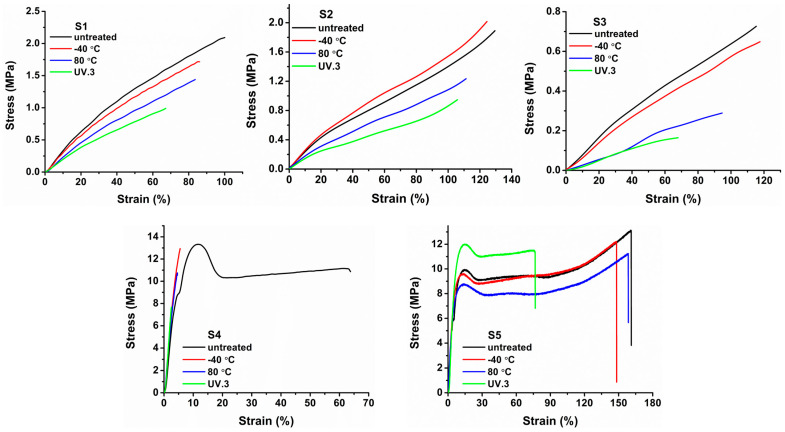
Stress–strain curves of the sucrose-based poly(ester-urethane)s: untreated, thermally aged (−40 °C and 80 °C), and UV irradiated (UV.3).

**Figure 8:**
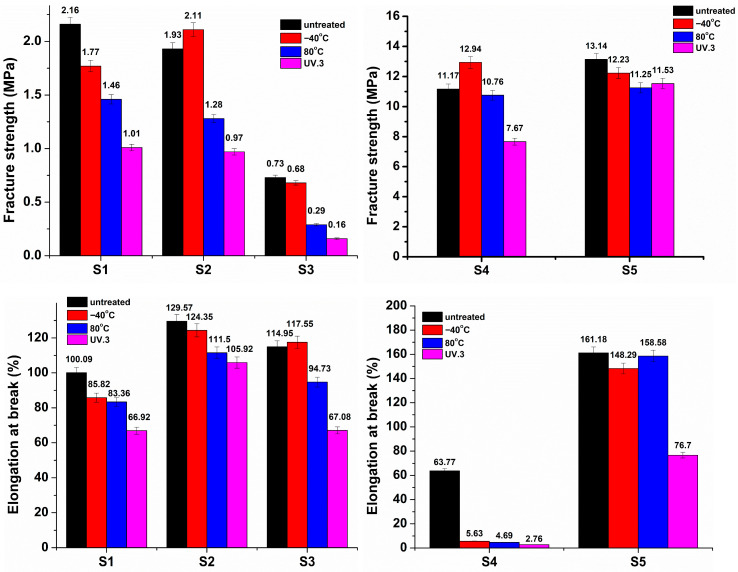
Fracture strength and elongation at break of the sucrose-based poly(ester-urethane)s: untreated, thermally aged (−40 °C and 80 °C), and UV irradiated (UV.3).

Stress–strain curves and the corresponding mechanical parameters, such as fracture strength and elongation at break, are presented in Figure 7 and Figure 8. S5 polyurethane has the highest fracture strength value (13.14 MPa (S5) vs. 11.17 MPa (S4), 2.16 MPa (S1), 1.93 MPa (S2), and 0.73 MPa (S3)) and elongation at break (161.18%). The use of polycaprolactone in the main chain (S5) imparts a higher degree of crystallinity in the polyurethane network, which is reflected in the high mechanical strength and elongation at break. Crystalline regions improve the mechanical properties in a manner similar to cross-linking [31]. S4 showed the lowest elongation at break (63.77%), due to rapid breakdown of physical cross-links upon elongation.

#### Mechanical Parameters After Thermal and UV Aging

Evaluating polymers’ mechanical characteristics is critical for understanding their behavior during aging processes. The evolution of the mechanical parameters after the aging process is depicted in Figure 8. Fracture strength and elongation at break showed a decreasing trend after thermal aging and three weeks of UV irradiation (UV.3). However, S4 exhibited the most dramatic decline, with the elongation at break reduced by more than 90%. In order to better understand the mechanical resistance of the synthesized polyurethanes after thermal and UV aging, the mechanical retention rates were calculated using the following equation [50], and the results are summarized in Table 4:

(2) $$Retention\;rate=\frac{S_{after\;aging}}{S_{untreated}}\times 100$$

where S stands for specific mechanical properties, which includes the fracture strength and elongation at break.

The retention rate of fracture strength reached 80–90% following cold treatment, whereas UV irradiation resulted in the lowest values, especially for the S1, S2, and S3 samples, which ranged between 20 and 50%. The substantial difference in the retention rate highlights that even if the mechanical properties are preserved after thermal treatment, UV exposure leads to important damage to the molecular structure. The retention rates for the elongation at break are higher than 60%, suggesting a good preservation of elasticity. However, an exception was observed for S4, where the elongation at break dramatically decreased after aging treatment, regardless of the condition used (thermal or UV). The use of PBA as a polyester in the synthesis of sucrose-based polyurethane led to a retention rate of the elongation at break between 4% and 8%. Such low values suggest increased brittleness of the polymer after aging treatment due to surface cracks and structural rearrangements [51], as evidenced by FT-IR spectroscopy. The S5 sample exhibited a stabilized profile for the elongation at break and fracture strength, confirming its resistance to UV exposure and both high and low temperatures.

### 3.5. Contact Angle and Surface Free Energy

**Figure 9:**
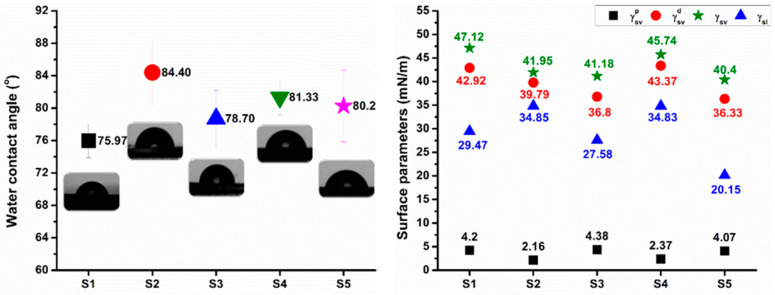
Water contact angles and surface parameters of the sucrose-based poly(ester-urethane)s.

The performance of the polymers under environmental exposure is strongly correlated with their wetting behavior. The contact angle, which is the edge angle of the liquid droplet in contact with the solid surface, is a frequently used technique to observe surface wetting. It reflects the energetic balance among the solid, liquid, and gas phases. Contact angles for the sucrose-based poly(ester-urethane)s are shown in Figure 9. The contact angle values showed minor differences as a function of the polyester type employed. The most hydrophilic surface was achieved for the S1 (75.97°) and S3 (78.70°) samples. Poly(ethylene adipate) and poly(diethylene glycol adipate) used in the synthesis of the polyurethanes increased the surface hydrophilicity. The highest value of the contact angle was achieved for S2 polyurethane at 84.40°. All obtained polyurethanes exhibited hydrophilic surfaces, which could accelerate their environmental degradability [52].

In order to obtain better insights into the chemical nature of the obtained polyurethane surfaces, surface free energy ($\gamma_{sv}$) and its polar ($\gamma_{sv}^{p}$) and dispersive ($\gamma_{sv}^{d}$) components were estimated using the Young (Equation (3)) [53], Owens and Wendt (Equation (4)), [54] and Fowkes equations (Equation (5)) [55]:

(3) $$\gamma_{sv}=\gamma_{sl}+\gamma_{lv}cos\theta$$

(4) $$\gamma_{lv}\left(1+cos\theta \right)=2\sqrt{\gamma_{lv}^{p}\gamma_{sv}^{p}}+2\sqrt{\gamma_{lv}^{d}\gamma_{sv}^{d}}$$

(5) $$\gamma_{sv}=\gamma_{sv}^{d}+\gamma_{sv}^{p}$$

where *θ* stands for the experimentally obtained contact angle values, $\gamma_{sv}$ and $\gamma_{lv}$ represent the surface free energy of solid/liquid in equilibrium with the saturated vapor of the liquid, $\gamma_{sl}$ is the interfacial free energy of solid to liquid, $\gamma_{sv}^{p}$ and $\gamma_{lv}^{p}$ are the polar components of the surface free energy, and $\gamma_{sv}^{d}$ and $\gamma_{lv}^{d}$ are the dispersive components of the surface free energy. Surface parameters were obtained using Milli-Q water (Millipore, ≥18.2 MΩ cm) and diiodomethane as wetting liquids, since the most trustworthy results require data from a polar–non-polar liquid pair [56]). The values are plotted in Figure 9.

Dispersion interactions were found to be the most influential in assessing the polarity of the obtained polyurethanes. Surface free energy values were higher than 35 mN/m, i.e., the synthesized sucrose-based polyurethanes are polar materials [57]. Surface free energy values were in good agreement with the contact angle measurements. The most hydrophilic polyurethanes (S1 and S3) are characterized by the highest values of the polar component of the surface free energy (4.2 mN/m and 4.38 mN/m, respectively).

#### Wettability After Thermal and UV Treatment

**Figure 10:**
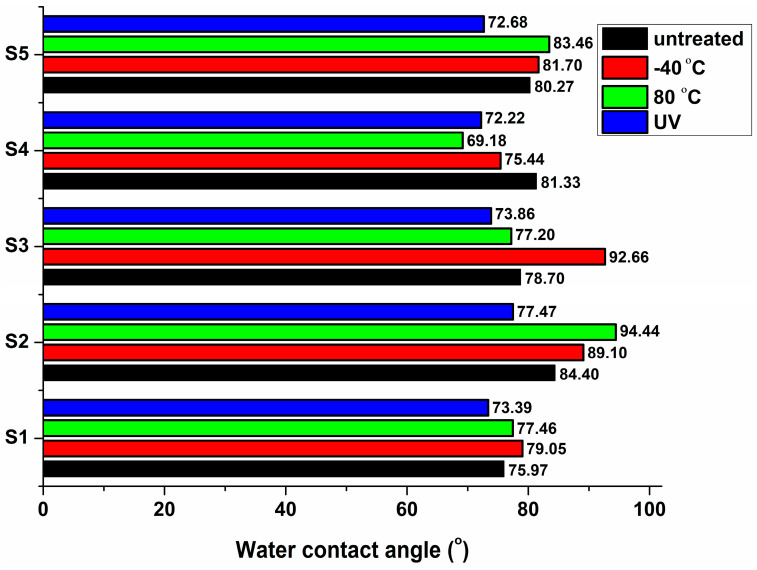
Water contact angles of the sucrose-based poly(ester-urethane)s: untreated, thermally aged (−40 °C and 80 °C), and UV irradiated (UV.3).

The evolution of the water contact angles after thermal and UV aging is illustrated in Figure 10. The water contact angle of sucrose-based polyurethanes increased slightly after thermal aging at low or high temperatures, indicating enhanced surface hydrophobicity due to molecular reassociation and rearrangement toward a more stable configuration [58]. The highest increase in the water contact angle was recorded for the S2 sample (94.44°) under high-temperature treatment and for the S3 sample (92.66°) under low-temperature treatment. S4 polyurethane became more hydrophilic following both thermal and UV aging, with the water contact angle ranging from 69° to 76°. These findings are consistent with the low mechanical performance, particularly elongation at break, as the polyurethane becomes brittle with increasing hydrophilicity. When comparing the two different aging processes, UV irradiation led to enhanced hydrophilicity in all samples, lowering the water contact angle to 73–78°. The increase in hydrophilicity could promote higher environmental degradability.

### 3.6. Surface Morphology Changes Induced by Thermal and UV Aging

**Figure 11:**
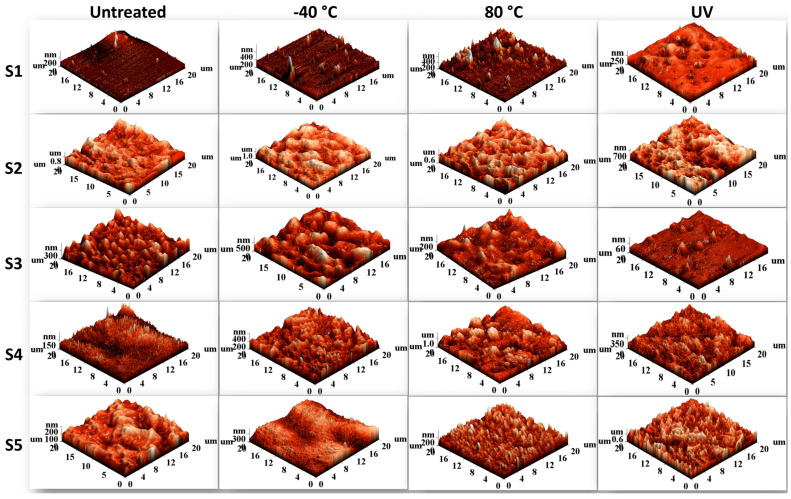
The 20 × 20 μm 3D AFM images of the sucrose-based poly(ester-urethane)s: untreated, thermally aged (−40 °C and 80 °C), and UV irradiated (UV.3).

The surface morphology was examined using AFM and SEM techniques. AFM provides information regarding microphase separation and domain morphology, since the hard and soft segments of the polyurethanes are well-highlighted due to their distinct contrast (light zones indicate hard domains, while dark zones indicate soft domains). The 20 × 20 μm 3D AFM images in Figure 11 show the surface morphology of polyurethane films in their untreated state and after high- and low-temperature treatment and UV irradiation. The values of the root mean square roughness (Sq) are inserted into Table 5.

The polyester diol type had a significant effect on the surface morphology of the obtained polyurethanes. Poly(hexamethylene carbonate) diol-based polyurethane (S2) had a higher roughness, regardless of the applied treatment. The root mean square roughness values were in the range of 130–208 nm, with higher values obtained after thermal and UV aging. Higher roughness was obtained for all samples after the aging process. However, there was an exception: the S3 sample showed a dramatic decrease in the S_q_ value of more than 90% after UV aging (from 80.04 nm to 7.07 nm), though the surface became less homogeneous. This behavior can be due to an increase in the cross-linking induced by UV light.

**Figure 12:**
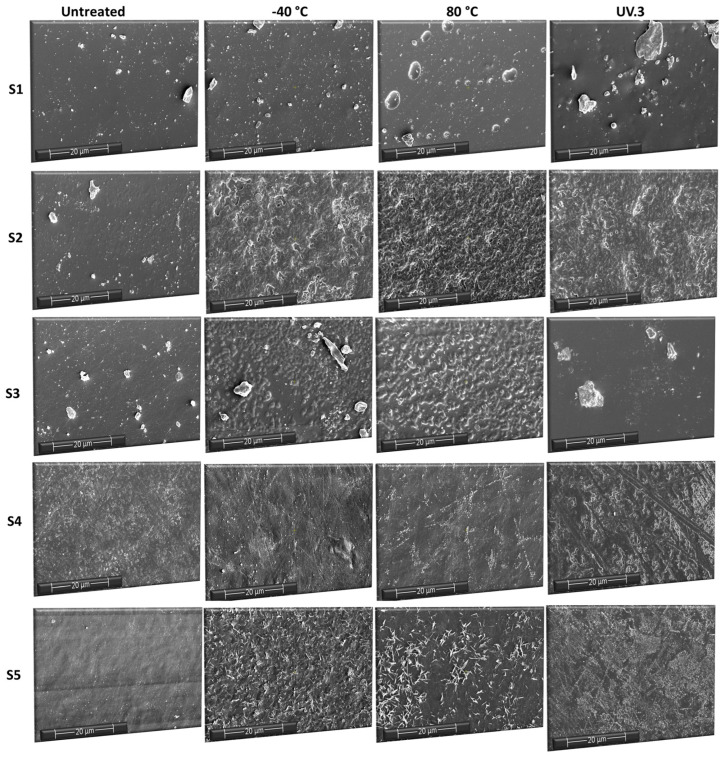
SEM micrographs of the sucrose-based poly(ester-urethane)s: untreated, thermally aged (−40 °C and 80 °C), and UV irradiated (UV.3).

SEM micrographs, obtained with the same magnification, of the pristine, thermally aged and UV-aged sucrose-based poly(ester-urethane)s were captured and are illustrated in Figure 12. Pristine films of all five samples exhibited smooth and homogeneous surfaces. Thermal aging (cold and hot temperature treatment) had a similar influence, leading to the deterioration of the polyurethane surfaces. The morphology became shriveled and wrinkled due to the appearance of small holes and cracks, especially for the S2, S3 and S4 samples. After UV irradiation, the S1 and S3 samples had a smooth surface, with no visible degradation. Instead, the surfaces of the other polyurethanes (S2, S4, and S5) had become nonhomogeneous and lumpy, with deep fissures, in accordance with the increased roughness. Surface roughness appears to result from random fractures and imperfections, marking the onset of polymer chain degradation.

### 3.7. Hydrolytic Degradation

The hydrolytic degradation of poly(ester urethane)s is well documented and involves the chain scission of the soft segments at the ester groups rather than at the urethane groups in the hard segments [59,60]. However, the introduction of sucrose units in the hard segments of the polyurethanes may influence this pathway [61].

**Figure 13:**
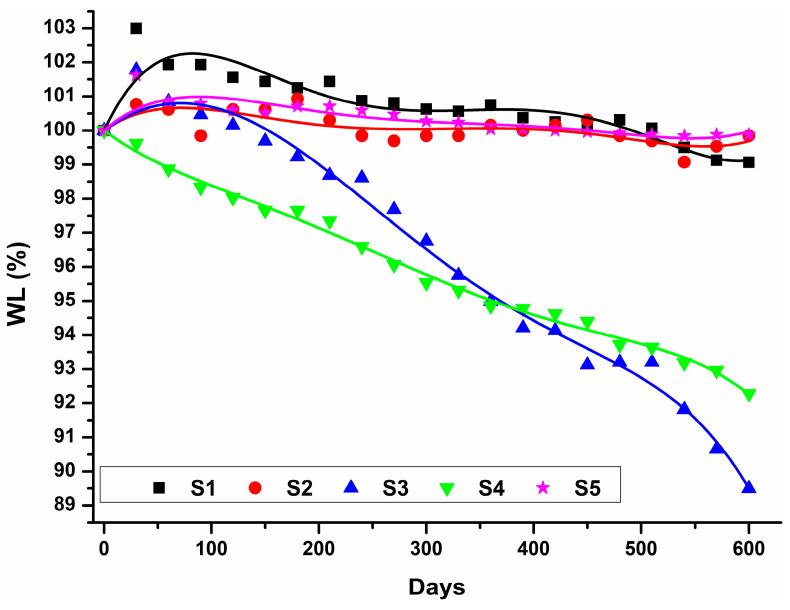
Variation of the weight ratio of the sucrose-based poly(ester-urethane)s as a function of the hydrolytic degradation time (the solid lines describe the overall trend of experimental data which are represented by symbols).

The variation in the weight ratio as a function of hydrolytic degradation time of the obtained sucrose-based poly(ester-urethane)s is illustrated in Figure 13. The S3 and S4 samples had a decreasing trend of the weight ratio after water immersion, reaching a value of 8–10% after 600 days of water immersion. Instead, the S2 and S5 samples had an almost linear evolution of the weight ratio, being stable in aqueous medium, even after almost two years. The weight ratio of the S1 sample increased throughout the first 100 days, indicating water intake.

Differences in aqueous behavior among the polyurethanes are due to the polyester composition of the soft segments, as the hard segments are identical. PHMC (S2) and PCL (S5) led to the best hydrolytic stability, which correlated well with the results in the previously reported literature [62]. Polyurethanes based on poly(adipate) diols (PDEGA (S3) and PBA (S4)) became brittle or sticky upon macroscopic inspection and underwent degradation through surface erosion [63].

The hydrolytic degradation process was monitored through FT-IR, SEM, and contact angle analyses of the water-immersed samples.

#### 3.7.1. Structural Modification During Hydrolysis

**Figure 14:**
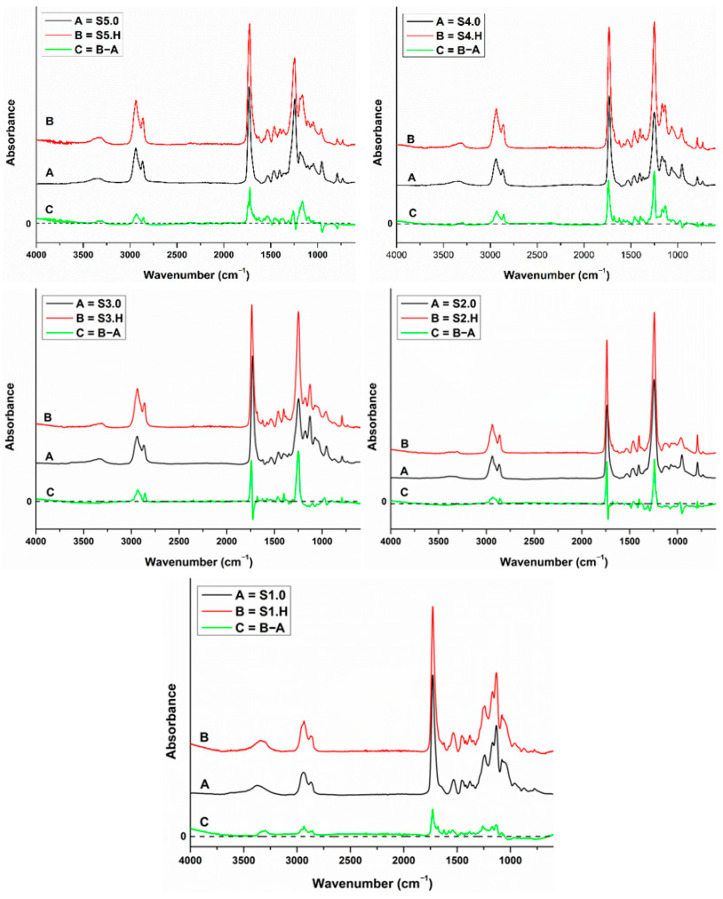
FT-IR spectra of the sucrose-based poly(ester-urethane)s before (S.0) and after a 600-day water incubation period (S.H) and the difference spectrum (C).

FT-IR spectra of the sucrose-based poly(ester-urethane)s before (S.0) and after a 600-day water incubation period (S.H) and the difference spectrum (C) are shown in Figure 14. No major structural changes were observed during hydrolysis and no new functional groups were generated. Ester groups are more susceptible to hydrolytic degradation followed by urethane groups. Difference spectra showed that the hydrolytically aged samples displayed stronger absorption bands than the pristine polyurethanes. The higher intensity of the absorption bands suggests enhanced hydrogen bonding and crystallinity after hydrolysis due to a chemicrystallization process that was previously observed on polyesters [64].

#### 3.7.2. Surface Analysis by SEM and the Contact Angle Technique

**Figure 15:**
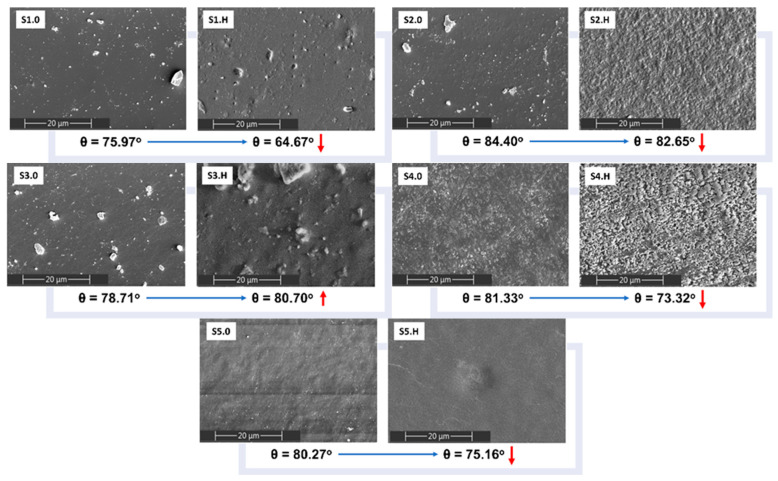
SEM micrographs and water contact angles (θ) of the sucrose-based poly(ester-urethane)s before (S.0) and after hydrolytic degradation (S.H).

The effect of the aqueous medium on the samples’ surfaces was investigated using SEM and the contact angle technique. The results are shown in Figure 15. SEM micrographs after the hydrolytic treatment indicate surface alterations, especially for the S2, S3 and S4 samples. There are significant changes in surface morphology, similar to the changes previously noted after thermal aging. This behavior is in good correlation with the weight loss ratio for S3 and S4 polyurethanes. Despite minor surface modifications, the S2 sample showed an almost constant weight during the hydrolytic process. The hydrophobicity of PCL segments limits the overall hydrolytic degradation of the obtained polyurethane (S5) and, therefore, the surface is less damaged [65].

The surface analysis was completed by contact angle measurements. Following the water incubation, the polyurethane surface exhibited increased hydrophilicity, as reflected in a decrease in water contact angles. An exception was observed for the S3 sample, where the contact angle increased slightly from 78.71° to 80.70°. Macroscopically, the surface became sticky, which led to the apparent increase in the water contact angle.

## 4. Conclusions

Structural and physico-chemical changes in sucrose-based poly(ester-urethane)s under thermal, UV, and aqueous conditions were analyzed. FT-IR and DSC spectroscopy confirmed preservation across all environments, showing no signs of new functional group formation. UV irradiation caused a sharper decline in mechanical properties than thermal aging. PBA-based polyurethane became brittle under all degradation conditions, showing the most severe loss, with the elongation at break reduced by over 90%. The decrease in the water contact angle correlated with mechanical deterioration, reflecting increased hydrophilicity upon degradation. Strong aging resistance was demonstrated for PCL-based polyurethane, which maintained over 85% of the fracture strength. AFM and SEM analyses revealed increased surface roughness after aging, accompanied by random cracks and flaws, marking the onset of early breakdown processes within the polymeric chains.

The polyester type strongly influenced hydrolytic degradation: PHMC- and PCL-based polyurethanes remained stable in aqueous medium, while PDEGA- and PBA-based polyurethanes lost 8–10% of their mass after 600 days of water immersion. Sucrose-containing PCL-based polyurethanes maintained performance under diverse degradation conditions.

## Figures and Tables

**Table 1:** The composition of the sucrose-based poly(ester-urethane)s.

Sample	Polyester Diol	Polyester Diol/HDI Molar Ratio	Polyester Diol/Sucrose Molar Ratio
S1	PEA	1/2	5/1
S2	PHMC	1/2	5/1
S3	PDEGA	1/2	5/1
S4	PBA	1/2	5/1
S5	PCL	1/2	5/1

**Table 2:** Thermogravimetric data for the sucrose-based poly(ester urethane)s.

Sample	Degradation Stages/Mass Loss (°C/%)			T_5%_ ^(a)^ (°C)	T_max_ ^(b)^ (°C)	Char Residue (%)
	I	II	III			
S1	280-395/25	395-470/69	470-520/2	320	441	3.35
S2	280-345/13	345-425/83	425-520/3	315	384	0.47
S3	280-345/8	345-480/89	480-520/1	321	447	1.44
S4	280-330/6	330-465/88	465-520/5	317	431	0.96
S5	280-345/7	345-470/88	470-520/4	328	430	0.74

^(a)^ Temperature corresponding to 5% mass loss; ^(b)^ temperature corresponding to the maximum rate of decomposition.

**Table 3:** Thermal parameters of the sucrose-based polyurethanes from the DSC scans obtained during the second heating run.

Sample		S1	S2	S3	S4	S5
T_g_ (°C)	untreated	−38	−42	−38	−43	−52
	−40 °C ^(d)^	−38	−41	−39	−46	−53
	80 °C ^(e)^	−37	−45	−44	−44	−53
	UV ^(f)^	−37	−40	−43	−44	−49
∆C_p_ (J g^−1^ C^−1^) ^(a)^	untreated	0.529	0.456	0.667	0.212	0.268
	−40 °C ^(d)^	0.531	0.426	0.589	0.203	0.244
	80 °C ^(e)^	0.180	0.623	0.619	0.288	0.214
	UV ^(f)^	0.538	0.488	0.709	0.150	0.236
T_m_ (°C) ^(b)^	untreated	-	-	-	50	41
	−40 °C ^(d)^	-	-	-	44	42
	80 °C ^(e)^	-	-	-	44	41
	UV ^(f)^	-	-	-	43	47
∆H_m_ (J g^−1^) ^(c)^	untreated	-	-	-	43.834	36.275
	−40 °C ^(d)^	-	-	-	43.951	35.146
	80 °C ^(e)^	-	-	-	59.742	36.850
	UV ^(f)^	-	-	-	31.468	35.878

^(a)^ Specific heat capacity, ^(b)^ melting temperature, ^(c)^ melting enthalpy, ^(d)^ aging at −40 °C, ^(e)^ aging at 80 °C, and ^(f)^ 3 weeks of UV irradiation.

**Table 4:** Retention rates after thermal and UV aging of the sucrose-based poly(ester-urethane)s.

Sample	Retention Rates After Thermal and UV Aging (%)					
	−40 °C		80 °C		UV	
	R_σ_ ^(a)^	R_E_ ^(b)^	R_σ_ ^(a)^	R_E_ ^(b)^	R_σ_ ^(a)^	R_E_ ^(b)^
S1	81.94	85.74	67.59	83.29	46.76	66.86
S2	109.33	95.97	66.32	86.05	50.26	81.75
S3	93.15	102.26	39.73	82.41	21.92	58.36
S4	115.85	8.83	96.33	7.35	68.67	4.33
S5	93.07	92.00	85.62	98.39	87.75	47.59

^(a)^ Fracture strength retention rate and ^(b)^ elongation at break retention rate.

**Table 5:** Values of the root mean square roughness (S_q_) of the sucrose-based poly(ester-urethane)s: untreated, thermally aged (−40 °C and 80 °C), and UV irradiated.

Sample		S1	S2	S3	S4	S5
Sq (nm)	untreated	17.40	137.90	80.04	29.71	27.51
	−40 °C ^(a)^	34.48	207.99	149.28	53.28	71.82
	80 °C ^(b)^	50.27	149.62	45.80	249.91	45.18
	UV ^(c)^	34.32	160.47	7.07	63.74	162.51

^(a)^ Aging at −40 °C, ^(b)^ aging at 80 °C, and ^(c)^ 3 weeks of UV irradiation.

## Data Availability

The raw data supporting the conclusions of this article will be made available by the authors upon request.
